# The entropic brain today

**DOI:** 10.1093/brain/awag206

**Published:** 2026-06-10

**Authors:** Robin L Carhart-Harris

**Affiliations:** Carhart-Harris Lab, Departments of Neurology, Psychiatry and Behavioral Sciences, Weill Institute for Neuroscience, University of California San Francisco, San Francisco, CA 94158, USA; Centre for Psychedelic Research, Imperial College London, London SW7 2AZ, UK

**Keywords:** entropy, entropic brain, complexity, criticality, fMRI, EEG, consciousness, psychedelics

## Abstract

Introduced in 2014 and revised in 2018, the entropic brain hypothesis has accrued a wealth of supportive evidence. The hypothesis states that—along a dimension of ‘the size of phenomenal consciousness’—expansive states reliably exhibit increased brain entropy, whereas the inverse applies for states of no or reduced consciousness. Examples of expansive states include expert meditation, flicker-light stimulation, near-death-like experiences, atypical breathing, REM sleep, the pre-ictal aura, unmedicated early psychosis and psychedelic drug states. Examples of states of no or reduced consciousness with low brain entropy, include disorders of consciousness, deep sleep, the anaesthetized state, seizure, post-stroke, ageing, cognitive impairment and neurodegenerative illness.

It is shown that the entropic brain has convergent, correlative, predictive, discriminative and external validity. Regarding its predictive validity, increased brain entropy under psilocybin (in a supportive context) predicts subsequent improvements in mental health (improved well-being 1-month post-dose). Regarding its discriminative validity, changes in brain entropy selectively index the ‘breadth of subjective experience’ versus alternative dimensions, such as arousal. Regarding portability/external validity, an entropy-related function is applied in generative artificial intelligence. In conclusion, the entropic brain is a useful model of conscious states.

## Teaser

Carhart-Harris reviews the evidence base for the entropic brain hypothesis, which links variations in conscious states to differences in brain entropy. He discusses the implications of the model for understanding consciousness and for guiding clinical approaches in psychiatry and neurology.

## Introduction

It’s a paradox, entropic phenomena are meant to decay over time, but not so the entropic brain hypothesis (EBH). Coined by the German physicist and mathematician Rudolf Clausius in 1865, the construct of ‘entropy’ has its origin in thermodynamics, the study of heat and energy. In that context, ‘entropic’ phenomena exhibit degradation or decay, as in the second law of thermodynamics, which states that (closed) systems—lacking a regular input of energy—tend towards increased disorder or ‘spreadoutness’ with time.^1^ Thermodynamic entropy—or the irreversibility of a process—has been applied to the action of psychedelic therapy for depression, where brain function moves closer to thermodynamic equilibrium after successful treatment,^2^ as per the ‘diffusion principle’—a principle of the second law.

The EBH, first introduced in 2014,^3^ and revised in 2018,^4^ is however, more directly concerned with entropy in the ‘information theoretic’ sense—applied to brain function—where it relates to the number of sufficiently independent ‘bits’ of spatiotemporal information exhibited by a property of spontaneous (i.e. on-going) brain dynamics. In this context—and in what follows—‘the entropic brain’ or ‘entropic brain effect’ refers to how properties of brain function become more unpredictable within expansive states of consciousness—typified by the psychedelic state.^5^ Within these expansive states, we can say that brain activity exhibits more independent bits of potential information, consistent with ‘entropy’ in the information theoretic sense, as first articulated by Claude Shannon.^6^ We use the qualifier ‘potential’ to acknowledge that the functional relevance of a signal must be decoded for it to be informative or meaningful to someone or something. The EBH says that—within a functionally relevant range—the degree of brain entropy within a given state of consciousness encodes ‘the felt breadth of that conscious state’. As entropy increases, so does the felt breadth of the subjective state, i.e. the state ‘feels’ expanded. Conversely, decreases in brain entropy correlate with reductions in conscious experience and, past a threshold, unconsciousness.

When introduced in 2014, the entropic brain hypothesis was initially inspired by insights gleaned from early human functional neuroimaging studies with the psychedelic drug, psilocybin.^7-9^ The hypothesis underwent an important update and revision in 2018,^4^ with the benefit of more human psychedelic imaging studies, data and evidence—affording greater clarity on which imaging modalities best detect the phenomenon and which properties of brain function—and analyses of them—are most sensitive to it. For example, it was found that EEG and magnetoencephalography (MEG) are especially useful modalities for detecting the effect in the temporal domain, and the data compressibility algorithm Lempel-Ziv complexity (LZc)— applied to spontaneous scalp or cortical potentials—is a particularly useful metric for quantifying the effect.^10^ Good mappings between the relevant EEG and MEG recorded brain states—and the LZc-computed effect—and corresponding subjective reports have since been shown, e.g. as demonstrated here for expansive states.^11,12^

Today, substantial evidence has accrued in support of the value, usefulness and validity of the EBH, such that it can now be regarded as a basic principle of conscious states. The principle is computational and ‘domain-non-specific’, in the sense that it applies across imaging modalities and metrics, biological systems and behaviour, and possibly even substrates. At an appropriate level or scale, applied to the dynamics of a system with sufficient complexity, ‘entropy’ functions as a bridging construct, translating across domains of physics, biology, behaviour and phenomenology. Translatability across domains implies that the principle has good portability, a matter that we shall return to later, e.g. in relation to artificial intelligence. Parallels can be drawn here with other bridging constructs within complexity science, such as self-organized criticality (and signatures of it) and the related notion of complexity itself.^13^

The purpose of the present article is to provide a necessary update on the supportive literature for the entropic brain principle, some of which has emerged since the key 2018 iteration of the EBH, and some of which predates it—but has since emerged as relevant. Some implications of the principle are discussed, including how it might guide our understanding of: (i) the nature of consciousness; (ii) therapeutic applications of psychedelic therapy; (iii) the nature of mental illness; and (iv) the action of effective treatments for it; as well as (v) the relevance of artificial general intelligence (AGI) to natural, human intelligence. Challenges to the model and its potential limitations are also discussed.

### Single versus multi-dimensional approaches to consciousness

Diagnoses and symptom severity measures of disorders of consciousness (DoC) have tended to adopt a single-dimension model of ‘consciousness’.^14,15^ Physiological and behavioural *responsiveness* to a controlled perturbation has been used as an operational method for defining and measuring ‘conscious level’.^16^ However, others have proposed multi-domain categorization schemes for consciousness.^17,18^ For example, distinguishing between ‘wakefulness’ versus ‘awareness’,^17^ ‘phenomenal’^19^ versus ‘access’ consciousness,^20^ or even more complex dimensions, including ‘perceptual richness’, ‘cognitive access’, ‘self-awareness’, ‘attention’ and ‘memory integration’.^18^

The EBH is intentionally simple. It does not deny that multiple dimensions of consciousness can be described,^18^ including dimensions relevant to the psychedelic state,^21^ yet it elects to focus on one specific aspect of ‘phenomenal consciousness’, namely its felt breadth or expansiveness. It shows that this dimension tracks closely with the entropy of spontaneous brain activity (ESBA) and spotlights specific properties of brain activity, such as population-level electrical fields. In other words, the current version of the EBH says that—within a relevant range of conscious states—‘the felt breadth of that state’ relates to the ESBA, e.g. the entropy of population-level electronic fields as indexed by EEG or MEG. In the psychological domain, rather than privileging behaviour, the EBH privileges subjective experience. This is one way in which it differs from other theories of consciousness, such as integrated information theory (IIT).^16^ Moreover, in contrast to IIT and the global neuronal workspace theory (GNW),^22^ the entropic brain does not present itself as an explanatory theory of consciousness, rather, it offers a (bridging) neurophysiological and phenomenological description of conscious states.

If framed in relation to a philosophical position, the EBH is most aligned with dual-aspect monism. Unlike hard materialism (or illusionism), it does not portray consciousness as an epiphenomenon caused by neuronal events. Rather, it offers a basic model of how a range of subjective states are encoded neurophysiologically and says that the relationship between the relevant subjective and neurophysiological phenomena is fundamentally ‘correlative’; also in contrast with hard materialism, it recognizes bidirectional causality between subjectivity and physiology rather than an exclusive one-way linear causation from brain to experience. Table 1 is intended to compare the EBH with other leading models of consciousness; this table is not intended to be exhaustive. Interested readers can consult prior work for more extensive comparisons of models of consciousness.^23^

**Table 1:** Comparison of EBH with other models of consciousness

Model or theory	Aim/purpose of model	Philosophical position	Psychological approach	Type 1 or 2 complexity
Entropic brain hypothesis (EBH)	A descriptive model of conscious states, with predictive validity	Dual-aspect monism	Equivalent importance ascribed to subjective experience and the encoding biology at the appropriate scale	Primarily type 1 but recognizes the relevance of type 2. Doesn’t yet commit to a specific measure of type 2 complexity
Predictive processing models of consciousness	Mostly descriptive models	Dual-aspect monism	Like EBH, looks to bridges between biology and psychology	Type 1 and 2 complexity
Integrated information theory (IIT)	An explanatory model of how consciousness is caused	Hard materialism and functionalism	Privileges function	Type 1 and 2 complexity
Global neuronal workspace (GNW)	An explanatory model of how consciousness is caused	Hard materialism	Privileges biology	Primarily type 2 complexity (integration)

Comparing the EBH with other leading models of consciousness by: (i) aims; (ii) philosophy; (iii) psychological approach; and (iv) relevance of type 1 or 2 complexity. See Seth and Bayne^23^ for a more exhaustive comparison of models of consciousness.

### Type 1 versus type 2 complexity

A useful distinction has been made between type 1 and type 2 complexity.^24,25^ Type 1 complexity is synonymous with information-theoretic entropy. It can increase monotonically to a maximum of pure randomness or ‘white noise’. In contrast, type 2 complexity incorporates structured complexity, such as signatures of self-organized complexity, like scale-free or fractal organization. A system or phenomenon can have very high type 1 complexity but no structure and therefore low type 2 complexity. Conversely, a system—such as one exhibiting self-organized criticality—can be high in both type 1 and type 2 complexity.

The EBH primarily relates to type 1 complexity. As we will discuss later, this is both a strength and weakness of the hypothesis. It is a strength in the sense that type 1 complexity is conceptually simple and straightforward to compute. As this article shows, data compressibility metrics like LZc work very well as biomarkers of the size of phenomenal consciousness. However, a model’s simplicity can also be its weakness, if, for example, it underfits the territory it is intended to represent. It is acknowledged that properties of type 2 complexity may be required for a fuller, more comprehensive account of consciousness.^26,27^ A recognition of type 2 complexity (i.e. criticality) has always been part of the EBH.^3^ However, while there are excellent type 1 complexity metrics for indexing conscious states (e.g. LZc on EEG or MEG data), the field has yet to discover an equivalently powerful measure of type 2 complexity that can index a salient and well-circumscribed dimension of consciousness with an equivalent (or superior) reliability. It is anticipated that this situation will change, and there is relevant work in this regard.^28^ Could information ‘synergy’ account for a dimension of consciousness variously referred to as ‘reflective awareness’ as it scaffolds on phenomenal consciousness? A proper discussion of this matter is beyond the scope of the present work.

### The origins of the entropic brain

Beginning chronologically, the initial inspiration for the EBH^3^ arose after using arterial spin labelling (ASL) functional MRI (fMRI) to examine the brain effects of the psychedelic, psilocybin. Under this drug, reductions in brain blood flow were observed in ordinarily hyper-perfused brain regions.^7^ This novel and unexpected finding motivated a re-consideration on the neurobiological action of psychedelic drugs in the human brain. The initial interpretation of the ASL results was thermodynamic in nature, namely that psilocybin had driven a process analogous to the diffusion principle—a classic illustration of the second law of thermodynamics. More directly, it was believed that psilocybin had caused a redistribution of perfusion towards a homeostatic equilibrium—a movement towards a more even distribution of perfusion—consistent with the tendency of (closed) systems to increase in entropy over time. The implication was the brain behaves more like a closed system under psychedelics, as would happen if a driving energy source was shut off.

Later, however, it became apparent that the perfusion decreases under psilocybin may represent an initial vasoconstriction^29,30^—and even a transient neurovascular decoupling. At the time, the observed decrease in blood flow caused some confusion regarding the general direction of metabolic^31,32^ and electrical changes in the brain under psychedelics.^33^ More plainly, rather than psychedelics decreasing brain activity, as the ASL result was originally interpreted, it is now apparent that the main effect of psychedelics is better described as one of ‘excitatory dysregulation’,^34^ especially in the cortex.

Supporting this updated interpretation, the first resting-state functional connectivity (RSFC) fMRI analysis of the action of a psychedelic (psilocybin), published alongside the perfusion result, suggested a functional desynchronization within the default-mode network (DMN) under psilocybin and an increase in coupling between the DMN and regions and systems that the DMN is ordinarily (relatively) functionally segregated from.^7,35^ Indeed, these initial suggestions of network ‘desegregation’ have since become a widely replicated finding across fMRI studies with classic psychedelic drugs,^12,36,37^ including in independent studies and designs,^38^ such that they are now, arguably, the best replicated fMRI-observed marker of the psychedelic state. A broadly similar profile now seems to apply for ketamine, at psychedelic-like doses.^39^

### FunctionalMRI and the entropic brain

While fMRI shone the first light on the entropic brain principle, different fMRI analysis metrics—portrayed as relevant to the EBH—have yielded somewhat inconsistent results. A variety of entropy measures have been applied to psychedelic fMRI data, but not all are comparably sensitive to the relevant basic phenomenon, this being an increase in the relative unpredictability of the dynamics of a property of spontaneous brain activity. One consequence of this is that inconsistent outcomes have been reported that have invited doubts regarding the reliability of the entropic brain principle.^40^ This would have been problematic for the principle if the examined fMRI metrics were equivalently sensitive, valid or optimal for indexing the phenomenon, but they are not.

Recent work has begun to clarify which fMRI metrics are sensitive to the entropic brain phenomenon. Functional MRI’s low temporal resolution implies a relative insensitivity to entropic effects in the temporal domain; but this modality’s high spatial resolution equips it with a special sensitivity to detect entropic effects in the spatial domain. Accordingly, metrics that leverage the high-spatial resolution of fMRI appear to be well-suited to detecting the entropic brain effect via this modality.

At least three promising fMRI metrics can be spotlighted in this regard, namely harmonic decomposition,^11,41-45^ normalized global signal complexity, or NGSC,^38^ and cortical spiral waves.^46^ Like certain metrics on electrophysiological data (e.g. LZc on EEG data), connectome harmonics have been used to categorize conscious states along an intentionally simple single dimension, often referred to as ‘conscious level’.^47^ Connectome harmonics are analogous to spatial patterns corresponding to standing waves that oscillate over a surface. They have been associated with the emergence of spatial patterns in nature, such as patterned animal skins, as well as ‘cymatic patterns’, where sound waves create complex symmetric spatial patterns in sand or liquid. The relevance of ‘Turing patterns’ to spatial harmonics has been noted^48^ as has their resemblance to visions of geometric patterns that are a quintessential feature of the psychedelic experience.^49^

Connectome harmonics are proving useful for characterizing conscious states and are relevant to the EBH. The EBH predicts a broader repertoire of harmonics under psychedelics alongside the emergence of higher spatial frequency harmonics. Focusing on relevant empirical work, a state associated with a negligible degree of consciousness—namely, a DoC without cue-specific brain responsiveness (fMRI–), was associated with predominantly low-complexity, coarse-grained spatial patterns within a resting-state timeseries, whereas a DoC with brain responsiveness (fMRI+) exhibited more entropic patterns within its timeseries. The anaesthetic, propofol, dose-dependently dropped the entropy of spatial harmonics (versus wake),^43^ while the psychedelic-like dissociative, ketamine,^43,50^ plus the classic psychedelics— psilocybin, LSD (lysergic acid diethylamide) and DMT (*N*,*N*-dimethyltryptamine)—all robustly elevated the entropy of spatial harmonics—and in a manner that correlated with the intensity and quality of the psychedelic experience,^11,41^ including the dynamic profile of DMT’s subjective effects across time.^51^ In a separate study, experienced meditators showed harmonic shifts resembling the high-spatial frequency harmonics of the psychedelic state, but only when actively within an intense mediative state.^44^ This result is consistent with the electrophysiology of meditative states, which is reviewed below.

Switching focus onto another spatial entropy metric applied to fMRI data, NGSC, interestingly and importantly, the stimulant methylphenidate failed to increase NGSC, whereas psilocybin robustly and reliably elevated it.^38^ Elevated NGSC under a psychedelic has also now been replicated via exceptionally deep-sampled fMRI data, pooling up to 9 h of psychedelic state data in several volunteers for a total of approximately 90 h. Increased NGSC was apparent in much of the brain but was especially pronounced in high-level association cortex, such as the precuneus, where the increases correlated with psychological insight scores.^52^ Further, the NGSC increases correlated with an LZc increase in EEG data from the same participants, providing the first cross-modality validation of the principle. Taken together, these results support the inference that increases in brain spatial entropy metrics on fMRI data are not merely indexing generic increases in arousal but rather something more specific,^38^ namely ‘the expansion of conscious experience’. In other words, the current version of the EBH proposes that observations of increased spatial or temporal entropy in macroscale brain dynamics under psychedelics encode increases in the ‘felt breadth of phenomenal consciousness’.^19^

Other entropy-related metrics applied to psychedelic fMRI data that have revealed a consistent increase in entropy under psychedelic drugs, include increased Shannon entropy under ayahuasca,^53^ Lempel-Ziv computed entropy rate on state transitions under LSD,^54^ Ising temperature under LSD,^55^ repertoire entropy under psilocybin,^8^ fractal dimension of activity and connectivity under LSD and psilocybin,^56^ perturbational complexity in a model of LSD fMRI data^57^ and sample entropy under LSD that predicted subsequent increases in the personality trait openness^58^—the latter being a well-replicated long-term psychological consequence of psychedelic use.^59^ Another study applied a variety of entropy metrics to a single resting fMRI data set and found increases in path-length distribution and sample entropy, as well as relationships between subjective effects and increases in Dynamic Conditional Correlation and LZc.^40^ A similar approach but using pooled data from various teams and studies involving a variety of psychedelics (such as these data)^37^—would offer a valuable extension of this work. One relevant effort that in this regard compared the fMRI effects of DMT, LSD, psilocybin and ketamine using a measure known as regional homogeneity, which looks at local synchrony/desynchrony.^60^ Consistent with the entropic brain principle, all four compounds decreased regional homogeneity, i.e. local desynchrony was increased—consistent with the NGSC findings described above.^38,52^

### Electrophysiology and the entropic brain effect under psychedelics

Perhaps the most significant breakthrough for the EBH occurred in 2017 with the publication of an important paper led by Michael Schartner and colleagues^10^ reporting increases in the entropy of MEG-recorded timeseries under three different psychoactive drugs, the dissociative, ketamine (at a dose that produces psychedelic-like effects), and the classic psychedelics, LSD and psilocybin. Schartner and colleagues applied the data compressibility algorithm LZc to resting-state MEG recordings of spontaneous brain activity. LZc has proved to be a particularly sensitive and reliable metric for indexing the entropic brain effect. LZc characterizes the diversity of sequences of signal or (potential) information. Like a dictionary or sequence of words or symbols in a passage of text, if a small number of words are repeated regularly, the passage’s entropy is low, but if each new word is different, then the entropy is high—and accordingly, the passage is rich in ‘potential information’—analogous to the content of a piece of classical literature (high entropy) versus the speech of someone learning a new language, repeating the few words that they have learnt (low entropy). LZc has proved to be a particularly powerful metric for computing the entropy of functional brain imaging timeseries, particularly those derived from EEG and MEG recordings.

Schartner and colleagues found a robust increase in LZc values for all three drugs as well as correlations between the increases in LZc and the subjective intensity of the drug-induced altered states. From this work, it was hypothesized that the increases in signal entropy reflects an increase in conscious level, consistent with the notion that psychedelics expand the breadth of phenomenal consciousness, i.e. what it is ‘like to be’ in any given conscious state.^19^ The insights afforded by this work motivated a revision of the EBH, published in 2018.^4^ This update spotlighted the superior temporal resolution of MEG and EEG timeseries—versus fMRI—and suggested that the application of LZc—and related metrics—on EEG and MEG timeseries may prove to be a particularly fruitful avenue for the EBH. This supposition continues to hold today.

Indeed, since that seminal paper in 2017, the same basic action of psychedelics (and relevant psychedelic-like drugs) on LZc-computed signal entropy has been replicated several times. Examples include two EEG studies with DMT,^12,61^ at least one with LSD,^62^ at least four with ketamine at psychedelic-like dosages,^50,63-65^ plus at least three with psilocybin.^66-69^ Some important details to highlight include the discovery of positive correlations between increases in LZc under DMT and subjective ratings of ‘richness of conscious experience’ as well as fMRI-measured increased global functional connectivity implicating how the ‘opening up’ of high-level networks correlates with the entropic effect seen in the EEG data (LZc).^12^ Moreover, in the above-mentioned deep-sampling psilocybin study, correlations were found between increased LZc in EEG data and NGSC in fMRI data—with both also correlating with psychological insight.^52^

At least one rodent study^70^ and one in cats^71^ have replicated the human EEG finding of increased LZc under sub-anaesthetic dosages of ketamine.^10^ Interestingly, both found an inverse-U relationship for ketamine dosage and brain entropy. Namely, brain entropy decreases when the dosage of ketamine is above a threshold for anaesthesia. This finding offers more compelling evidence for the principle that brain entropy maps to conscious level in its simplest sense. Entropy decreases when the dosage of ketamine is sufficiently high to cause unconsciousness.

Some studies have applied other signal entropy metrics to electrophysiology data and found comparable effects to those seen with LZc. It is now well recognized that the LZc algorithm is poorly suited to non-stationary timeseries, such as those associated with rapid onset psychedelics such as DMT and 5-methoxy-DMT (5-MeO-DMT). Better suited algorithms for tracking non-stationary dynamics include the ‘complexity by sample entropy rate’, or ‘CSER’ metric,^72,73^ and permutation entropy, as nicely demonstrated by Lewis-Healy *et al*.^74^

Literature on the effect of classic psychedelics on the entropy of local field or scalp potentials in non-human animals is surprisingly sparse. More work is needed to fill this knowledge gap, particularly given the apparent relevance of brain entropy to the breadth of phenomenal consciousness in humans—and, just as importantly, clinically relevant mental health outcomes.^66,68^ Evidence for the clinical relevance of anatomical neuroplasticity effects observed in non-human animals^75^ would carry greater translational appeal if they were found to relate to entropic electrophysiology or molecular signalling under the drug. See this relevant work.^76,77^ It is important that preclinical research not be divorced from findings and models that are proving to be useful in humans.^78^

## Other psychoactive compounds and psychedelic microdosing

Reduced brain entropy has been seen during drowsiness induced by the deliriant scopolamine at psychoactive doses,^79^ consistent with a wealth of data from several studies showing drug-induced reductions in brain entropy with a range of anaesthetics,^16,71,80-85^ sedatives^65,86^ and antipsychotics.^87^ Like the dissociative ketamine (at psychedelic-like doses), nitrous oxide (N_2_O)—which shares a somewhat related glutamatergic pharmacology to ketamine—increased LZc on resting-state EEG, albeit in rats, but only during an onset phase, after which LZc subsequently decreased. Presumably the later-phase decrease in LZc occurred with a higher plasma concentration of drug that caused some loss of consciousness;^70^ however, decreased signal complexity has been seen with a low-dose of N_2_O in humans,^88^ implying that it does not act like a typical psychedelic.

An interesting low-dose LSD EEG study found the expected dose-dependent rise in brain entropy with escalating doses of LSD. Subjective effects were felt at a maximal dose of 26 μg, which is higher than the psychedelic-dose-threshold for a (sub-perceptible) ‘microdose’ of LSD. The increase is brain entropy was highest at this maximal dose. Importantly, in the same study, the potently psychoactive cannabinoid tetrahydrocannabinol (THC) and the stimulant methamphetamine failed to increase LZc to a significant degree.^62^ Two other studies with low-dose psilocybin (one with psilocybe mushrooms) failed to see an entropic brain effect, but in both cases, the dose was so low that psychoactive effects were unreliably reported.^66,68,89^ One prior study with THC found a dose-dependent increase in LZc that correlated strongly with positive psychotic symptoms, including disorganized cognition.^90^ This finding aligns with the psychedelic-like phenomenology of the sufficiently high-dose THC or high-potency cannabis experience and usefully highlights the valence non-specific quality of expanded states, i.e. these states can feel euphoric and/or dysphoric, potentially related to their ability to increase psychological plasticity, therefore creating highly context-dependent effects, as discussed here.^91-93^

Taken together, these pharmacological data imply a somewhat selective relationship between increased brain entropy and the experience of consciousness expanding. This relationship is reliably seen with sufficiently high doses of prototypical psychedelics, like LSD, psilocybin and DMT (Table 2) and can be seen with psychedelic-like compounds, such as ketamine, at relevant dose ranges.^71,90^ Work is currently underway to assess how the atypical psychedelic, 5-MeO-DMT, affects brain entropy, with some published^95^ and preliminary work suggesting that this compound does indeed increase brain entropy—and perhaps markedly so. Both studies report increased high frequency activity—known to account for most of the entropic brain phenomenon,^72, 73^ but the emergence of low-frequency ‘slow waves’ (<1.5 Hz) complicates the picture. Emergent slow waves under 5-MeO-DMT could relate to the loss of ego-function/reflective awareness and associated amnesia that can occur with high doses of 5-MeO-DMT.

**Table 2:** Evidence for the EBH

Decreased brain entropy with reduced or absent consciousness	Elevated brain entropy in expanded conscious states
Anaesthetized or sedated state^16,42,43,45-87,94^	Psychedelic state^3,4,8,10-12,36-45,50-62,65-74,86,95,98,99^
Ketamine as an anaesthetic^70^	Psychedelic state versus sedated state^86,100,101^ and DoC^102,103^
NREM sleep^71,87-92,104-109^	Ketamine as psychedelic-like^43,50,63-68,71^
Disorders of consciousness (DoC)^16,43,81,110-114^	Highly immersive states of meditation^43,44,119-124^
Mild cognitive impairment^125-127^ and neurodegenerative disorders^128-135^	REM sleep^71,104,105,108,109,136,137^
Antipsychotic drug effect^87^	Flicker-light^138,139^
Mid-seizure (lost consciousness)^140-142^	Atypical breathing^143,144^
Postoperative drowsiness/delerium^145,146^	Near-death-like experience^147^
	Musical immersion,^148^ creativity,^149-154^ divergent thinking,^152^ fluid intelligence,^155^ the state of awe^156^
	Pre-ictal aura of temporal lobe epilepsy (dreamy state)^157^
	Recovery of consciousness after DoC and anaesethsia^82,86,100,101,113^
	High emotionality,^158^ including states of panic and stress,^159^ awe^156^ and mania,^160,161^and early or acute psychosis^87,87,162-164^

Evidence for the EBH as it applies to conscious states at either end of the consciousness spectrum, i.e. states of absent or reduced consciousness versus states of expanded phenomenal consciousness. These findings show the depth and breadth of evidence for the convergent validity of the EBH. Included within this table are several studies that also support the correlative validity of the EBH, e.g. that increased ESBA correlates with relevant phenomenology^12^ and functional MRI-measured ESBA correlates with EEG-measured ESBA (with both also correlating with the relevant phenomenology). EBH = entropic brain hypothesis; NREM = non-REM sleep; ESBA = entropy of spontaneous brain activity.^52^

### Sleep and dreaming

As reviewed above, psychedelic drugs appear to robustly and reliably increase brain entropy, especially when indexed via metrics like LZc or CSER applied to EEG or MEG data or NGSC or connectome harmonics on fMRI data; but what about other examples of non-ordinary states where the breadth or richness of conscious experience has also been said to increase versus a relevant baseline?

Examining sleep, several electrophysiology studies across non-human animals^71,104,105^ and in humans^106-109^ have shown decreased signal entropy in deep, non-REM (NREM)-sleep versus wake, where conscious awareness is typically absent. Other studies have shown an increase in brain entropy in REM-sleep versus NREM-sleep,^71,104,105,108,109,136,137^ as one would expect given the richer and more expansive quality of consciousness in REM-sleep dreaming versus deep, dreamless sleep, as is typical of NREM-sleep.

Curiously, psychedelics given just before^96^ or after sleep onset^97^ have been found to promote aspects of REM-sleep, and dreaming too, possibly because of the tendency for the psychedelic to increase brain entropy—therefore biasing brain physiology in the direction of REM-sleep and dreaming. Further, natural language processing has found similarities between dream—and psychedelic trip—reports,^165^ and other work has spotlighted phenomenological and neurophysiological similarities between the psychedelic and (REM-sleep) dream state.^115,116^ Indeed, some atypical psychedelics, such as ibogaine, have been called ‘oneirogenic’—which translates as ‘dream-generating’, and one interesting—but as yet unverified—hypothesis has proposed that the endogenous psychedelic, DMT, is released in the brain during REM-sleep, thereby inducing the dream experience.^117^ Regardless of the validity of this hypothesis, the idea that the psychedelic state mimics REM-sleep physiology, akin to a (hybrid) waking-dream-state, has history.^115^ There is a common suppression of activity within midbrain serotonin cell bodies in REM-sleep and the psychedelic state,^118,166,167^ and the disinhibition of other neurotransmitter systems such as the acetylcholine, dopamine and glutamate systems may also be shared across these states. Moreover, high-frequency gamma oscillations are a common feature of both REM-sleep and the psychedelic state.^168^ Like psilocybin,^98^ DMT has been found to promote high-frequency gamma oscillations in the rat cortex.^99^ Increased high-frequency activity is a major carrier of the entropic brain effect.^72,73^ More work is needed to assess whether ibogaine increases brain entropy, but given its phenomenology, and the assumptions of the entropic brain model, it can be speculated that it does.

### Non-drug-induced increases or elevations in brain entropy

Aside from the association with REM-sleep and dreaming, as just described, increased brain entropy has also been observed in experienced meditators;^119-122^ particularly during expansive conscious states, such as those described as ‘Jhana’^122^ or ‘cessation’ states,^121^ see here for a relevant review.^123^ Of note, it’s important not to conflate states of expanded phenomenal consciousness—induced via meditation—with relaxation, fatigue, restlessness or frustration states that are also common in meditators, particularly in novice meditators, see^169^ for a relevant discussion. Conflating distinct meditative states with each other could account for inconsistencies within the meditation EEG and MEG literature.^123^

Some other non-ordinary states of consciousness in which brain entropy has been found to increase, include flicker-light experiments, where strobe lighting, e.g. at the alpha frequency, can drive closed-eye visual images or ‘hallucinations’ that have a subjective quality not dissimilar to those of the psychedelic drug state.^138^ There is some preliminary evidence that alpha-frequency flicker can drive the emergence of harmonics at higher frequency multiples of the entraining frequency. One interpretation of this apparent induction of higher frequency harmonics via flicker is that they relate to the emergence of eyes-closed geometric hallucinations or visions. Further work is needed to test this hypothesis, but it would align with some recent optical imaging work in rodents exposed to flicker,^139^ as well as local field potential recordings in mice given psilocybin, where high-frequency (gamma) oscillations emerged during peak behavioural effects of the psychedelic.^98^

Like flicker-light, it has long been known that atypical breathing can induce non-ordinary states that resemble, in certain respects, the phenomenology of the psychedelic drug-induced state.^143^ Some recent EEG work found increased brain entropy shortly after an atypical breathing paradigm referred to as ‘breathwork’, as well as a correlation between the entropic state and ratings of psychedelic-like subjective phenomena induced by the breathing method.^144^

To our knowledge, there are currently no reports of brain entropy analyses performed on data derived from conditions of sensory deprivation or so-called ‘dark retreat’ settings—another method for inducing psychedelic or psychedelic-like states of consciousness.^170^ See the pivotal mental states model (PiMS) for speculations on the physiology, pharmacology and phenomenology of such states, including how they may arise and for what function. The PiMS model offers an account of a naturally evolved system for elevating brain entropy and plasticity. Psychedelic drugs hijack this system to elicit their effects, pushing plasticity to supranormal levels. In brief,^92^ the PiMS model says that entropic, hyper-plastic states arise under conditions of adversity or stress for the purpose of catalysing rapid, deep and potentially lasting psychological change. It is proposed that this happens for adaptive reasons, e.g. to enable an organism to overcome potentially life-threatening challenges. Further supporting this hypothesis, experimentally induced stress has been shown to increase brain entropy.^159^ Moreover, high emotionality,^158^ manic states^160,161^ and states of awe^156^ have been associated with increased brain entropy. The relationship between brain entropy and plasticity is discussed here.^93,171^

Other examples of non-ordinary states exhibiting increased brain entropy and psychedelic-like phenomenology, include the near-death-like experience,^147^ the experience of listening to improvised versus classically scripted music^148^ and the pre-ictal aura of epilepsy;^157^ indeed, in relation to the pre-ictal state, see this work on the phenomenology and neurobiology of the ‘dreamy state’ of temporal-lobe epilepsy, including states induced by medial temporal lobe stimulation, and their parallel with other psychedelic or ‘dream-like’ states.^115^

Elevated brain entropy has been observed in early, unmedicated psychosis, with positive symptoms,^87,162-164^ a picture that reverses as the disorder develops towards negative symptoms and sedation via chronic medication.^87^ Parallels have been drawn between the phenomenology of early psychosis and the psychedelic drug state^92,115^ but these resemblances tend to lose fidelity as the disorder morphs over time. Chronic medication and the impact of the illness tend to change the individual’s presentation, accounting for a progressive divergence away from psychedelic-like states. See this relevant work in this regard.^172^

### Disorders of consciousness

As exemplified by sleep and anaesthesiology research, it has long been known that brain entropy decreases in parallel with a reduction in conscious level. Entropy measures applied to EEG have long been used to track the depth of anaesthesia.^82,85,94^ Closely aligned with this, it is known that individuals suffering from disorders of consciousness, such as coma, unresponsive wakefulness syndrome (i.e. the ‘vegetative state’) and the minimally conscious state, show low brain entropy.^110-114^ Indeed, the low level of brain entropy in these disorders relates to the severity of the disorder, such that it can be used to categorize the disorder and inform on how it should be diagnosed.^16,43,81,110^ This powerfully illustrates the broad applicability of the EBH—beyond a mere focus on psychedelic drug states. Indeed, in a longitudinal EEG study, individuals who emerged from a disorder of consciousness—recovering some degree of consciousness—showed a corresponding increase in brain entropy as their condition improved,^113^ endorsing the principle that the magnitude of brain entropy parallels an enrichment or expansion of consciousness.

Inspired by this principle and the observation that brain entropy is pathologically low in disorders of consciousness yet reliably elevated via psychedelics, it was proposed that psychedelics might treat disorders of consciousness.^47^ Work is currently underway to test ketamine^102^ and psilocybin for this purpose. Some biologically informed computational modelling supports the hypothesis,^173^ and a single case study exists of an uncontrolled experiment involving psilocybe (‘magic’) mushrooms administered to a middle-aged female in a minimally conscious state. Novel behaviours, such as never-seen-before bilateral leg-lifts were observed, and an increase in brain entropy was seen that tracked with the typical profile of subjective effects. However, no improvements in standard assessments of conscious level were observed, either acutely or sub-acutely, thereby tempering optimism for the hypothesis.^47,103^

Indirect support for the idea that psychedelics can improve conscious level in disorders of consciousness does exist, however. For example, increases in brain entropy were observed when psychedelics were introduced over a baseline of pharmacologically reduced conscious level, both in rodents^100^ and humans.^86,101^ One critical angle to consider here is that the EBH relates most closely to ‘phenomenal consciousness’,^19^ i.e. ‘subjective experience’, and even more specifically, its felt size or breadth. As implied by the case report of psilocybe mushrooms given to an individual in a minimally conscious state,^103^ the hypothesized enrichment of subjective experience could occur with this intervention, but in the absence of an adjacent improvement in reflective awareness or ‘access consciousness’.^20^ The failure of (a hypothetical) increase in phenomenal consciousness translating into an adjacent improvement in a behavioural index of conscious level could both: (i) add validity to the two-component model of consciousness (i.e. ‘phenomenal’ versus ‘access’); and (ii) imply that greater effort is needed to achieve the translation from one (phenomenal) to the other (access). For example, better outcomes might be achieved via experimenting with dosage parameters and adjunctive neurorehabilitation.^103^ Clearly, there are complex ethical matters to consider here.^47^

### Neurological and neurodegenerative disorders, cognitive impairment and ageing

The idea that a loss of physiological complexity is a feature of ageing has a long history, dating back at least to the early 1990s^174^ and work on the relevance of this principle to neurophysiology has received a recent update.^175^ There is some evidence to support the hypothesis;^176^ however, the evidence is also mixed,^177^ perhaps due to a lack of within-subject longitudinal data examining putative changes over a sufficiently long period of time. The felt breadth of phenomenal consciousness does not obviously decrease as a function of age—while faculties of reflective awareness do become impaired in neurodegenerative disorders, including dementia, and there, entropy decreases as expected.

Without the necessary longitudinal data, the field has been left to rely (mostly) on cross-population comparisons, which are methodologically weak. As alluded to above, when examining brain pathologies of ageing and especially neurodegenerative disorders, the picture is clear and consistent. For example, brain entropy is low in individuals with Downs syndrome with mild cognitive impairment (MCI)^125^ and in MCI more generally.^126,127^ The literature on brain entropy changes in Alzheimer disease (AD) is clearer still, e.g. with a large number of studies showing reduced brain entropy,^128-135^ including in a way that reliably diagnoses the classification of AD versus health^130,132^ and MCI versus AD.^131,132^ Brain entropy has also been shown to be low mid-seizure, during the loss of consciousness,^140,141^ as well as in a drowsy state of postoperative delirium.^145,146^

Taken together, at both poles of the phenomenal-consciousness-spectrum, the one associated with unconsciousness—and the opposite extreme associated with highly expansive states, the evidence for the EBH is compelling; see Table 2.

### Other aspects of entropic physiology and behaviour

No organ is an island ‘entire of itself’, including the brain. What is known of the entropy of other aspects of physiology and behaviour? Less entropic cardiac activity^178,179^ and breathing^179^ has been observed with ageing, whereas more entropic cardiac activity has been observed under psychedelics, associated with subsequent improvements in mental health outcomes, leading to a coining of the phrase ‘the entropic heart’.^180^ The content of spoken language was found to be more entropic under psychedelics than in normal waking consciousness, an effect described as ‘the entropic tongue’.^181^ Given the relative ease of recording language and peripheral physiology, there is great scope for examining how each relates to psychedelic and other conscious states, and how this can inform on health. This topic is too expansive to be examined within the present paper but see^182^ for one relevant example.

The topic of entropic activity in behaviour and other domains of physiology is relatively underdeveloped. More work is needed to confirm which domains of physiology and behaviour best parallel the entropic brain effect and which do not.

### Entropy and creativity

Theoretical work has proposed an association between more entropic computation and creativity.^149^ Consistent with this idea, one study found more entropic hand movements in those delivering more creative solutions in a creativity paradigm,^150^ while another found more entropic melody choice in those engaging in creative musical improvisation.^151^ More directly relevant to the EBH, higher entropic brain activity (fMRI) has been found to correlate with general intelligence,^183^ fluid intelligence,^155^ divergent thinking^152^ and creative insight.^153^

We note an alignment between theories linking entropy, creativity and insight,^149,154^ the above-cited fMRI findings,^152,153,155^ and findings of an association between increased brain entropy under psychedelics and psychological insight.^66,68^ One interpretation is that more ‘bits’ of potential information carried by the brain’s spontaneous neurophysiology implies more (potentially useful) content to be deciphered, processed, integrated and applied. This perspective aligns with the etymology of ‘psychedelic’ itself, which means ‘revealing the psyche’^5^ as well evidence that psychedelic therapy aids psychological growth through processes of psychological insight.^184,185^ Indeed, this interpretation is closely aligned with the EBH itself, and the dual-aspect monist perspective, where phenomenology runs parallel to the relevant neurophysiology, and mental phenomena have causal power.

Relatedly, the ethos and quest of the EBH is to find relevant (brain) code(s) that encode the relevant subjective experience(s): good maps for the relevant territory: subjective experience. Briefly, it can be speculated that neural encodings may prove to be more elusive across brains than ‘within’ any given brain,^186^ underlining the challenge of finding a ‘common code’.

### Re-analysing the psilocybin and LSD MEG data

Let’s now examine some of the nuances of the entropic brain principle. There have been novel re-analyses of the same MEG data included within the seminal paper by Schartner *et al*.^10^ One utilized the factorial design of the LSD MEG study to examine the relative size of the entropic brain effect (versus normal waking consciousness) under conditions of differing sensory stimulation, namely quiet eyes-closed rest, versus eyes-closed with music, versus eyes-open with movie-watching plus sound. Results revealed that—although greater sensory stimulation tends to elevate brain entropy (including under placebo)—the drug-induced entropic brain effect shows the greatest differential (versus placebo) when sensory stimulation is most minimal, i.e. in the quiet, eyes-closed resting condition.^187^ Similar results were found in a recent psilocybin fMRI study that found smaller effect sizes of drug (psilocybin) versus placebo on metrics of brain activity change when participants were asked to carry out a perceptual paradigm. In contrast, effect sizes were extremely large for the task-free resting-state condition.^38^ Other work has tended to reinforce the view that goal-directed cognition decreases brain entropy, while sensory complexity tends to increase it.^188,189^

Recent work finessing entropy measures on EEG data has shown that the effect is carried disproportionately by high-frequency activity, particularly within the gamma range.^72,73^ Some recent mouse work,^98,99^ and previous connectome harmonic findings with psychedelics and fMRI^45^ have implied that psychedelics do not induce a ‘white-noise’-like state of unstructured chaos, but rather a quality of brain activity that is ‘finer and faster’—manifesting as increased information theoretic entropy in the spatial and domaintemporal. This implies that there is a (hidden) structure to brain activity in the psychedelic state, aligned with increased type 2 complexity (and self-organized criticality). This structure is expressed within higher-than-normal temporal and spatial frequencies. This should motivate researchers to ensure that their recording and data-filtering methodologies are sufficiently sensitive to detect the (hypothesized) ‘faster and finer’ effects. More specifically, low-pass filtering may blind spot researchers to an entropic brain effect contained within faster but filtered-out data.

Regarding self-organized criticality (and type 2 complexity), one analysis examining electrocorticography (ECoG) and MEG data found increases in LZc under LSD but also drops in entropy during seizure and with propofol.^142^ This same publication reported evidence of greater brain criticality (type 2 complexity) with the psychedelic, consistent with an early prediction of the original EBH.^3^

## Closer to—or beyond—criticality?

The topic of self-organized criticality and its relevance to conscious states is complicated but important. Self-organized criticality refers to complex dynamical systems that exhibit the emergence of complex properties (type 2 complexity) when their dynamics are positioned within a critical zone between the extremes of frozen order and pure chaos (white noise). In either direction outside of this critical zone, (type 2) complexity is compromised, yet within it, properties such as long-range correlations (i.e. correlations spanning the entire system), slowness to recover (from even small perturbations—as per the ‘butterfly effect’), a large repertoire of sub-states and scale-free, power-law-observing dynamics are evident. These are signatures of type 2 complexity or criticality.

There have been several influential papers on the topic of self-organized criticality, brain function^190^ and consciousness; a small cross-section is cited here.^191-193^ See also this work linking in psychedelics^194^ and exceptional meditative states, i.e. jhana (absorption) states. The original 2014 entropic brain paper hypothesized that—during normal waking consciousness—brain dynamics resides just shy of full-blown criticality. By implication, there is scope for the brain to exhibit stronger signatures of criticality and complexity during psychedelic higher entropy states.^41,45,54,56,142^ Other work has, however, suggested that psychedelics dial brain activity beyond a critical regime into a state of compromised structure, i.e. increased type 1 complexity but compromised type 2 complexity.^55,195^

Opinions vary as to the correct nomenclature for an entropic regime that surpasses a critical threshold, transitioning into disorder; is such a regime ‘sub’ or ‘super’ critical? Accordingly, it seems sensible to avoid committing to either prefix but rather to clarify: (i) what one’s operational definition of criticality is, i.e. which properties of brain function are being regarded as ‘critical’ and via what metric; and (ii) does the experimental condition or intervention appear to increase or decrease the relevant signature of criticality?

Clarifying these questions will help advance the debate regarding whether psychedelics^41,54,56,142^—and other related states, such as expert meditation^122,124^—increase criticality or push brain dynamics beyond a critical zone into a disordered regime where (type 2) complexity is compromised.^55,195^ It feels appropriate to conclude that psychedelics can both increase (certain) signatures of criticality^45,142^ and yet decrease others;^196^ the key consideration being: the relevant signature and what it relates to functionally, e.g. in relation to a well-defined dimension of consciousness. Efforts are ongoing to answer important questions, such as whether certain properties of criticality (type 2 complexity) relate to certain specific aspects of conscious functioning. Accordingly, it feels advisable not to unconditionally commit to either principle, i.e. that psychedelics dial brain activity closer to—or beyond—criticality. Answers to relevant important questions are likely to be dependent on the specific nature of definitions and contexts.

Regarding the question of relationship between the psychedelic state and brain criticality, we should also consider the role of drug dosage and the specific psychedelic in question. For example, the subjective character of the high-dose 5-MeO-DMT experience is such that it is tempting to speculate a surpassing of a critical point—into a regime that is too entropic for type 2 complexity to be preserved, as implied in Fig. 1. Examining this and related questions could shed new light on principles of brain function and conscious experience, for example, revealing what aspect(s) of brain function are lost when ego consciousness temporarily disappears, as occurs quite reliably with high-dose 5-MeO-DMT.

**Figure 1:**
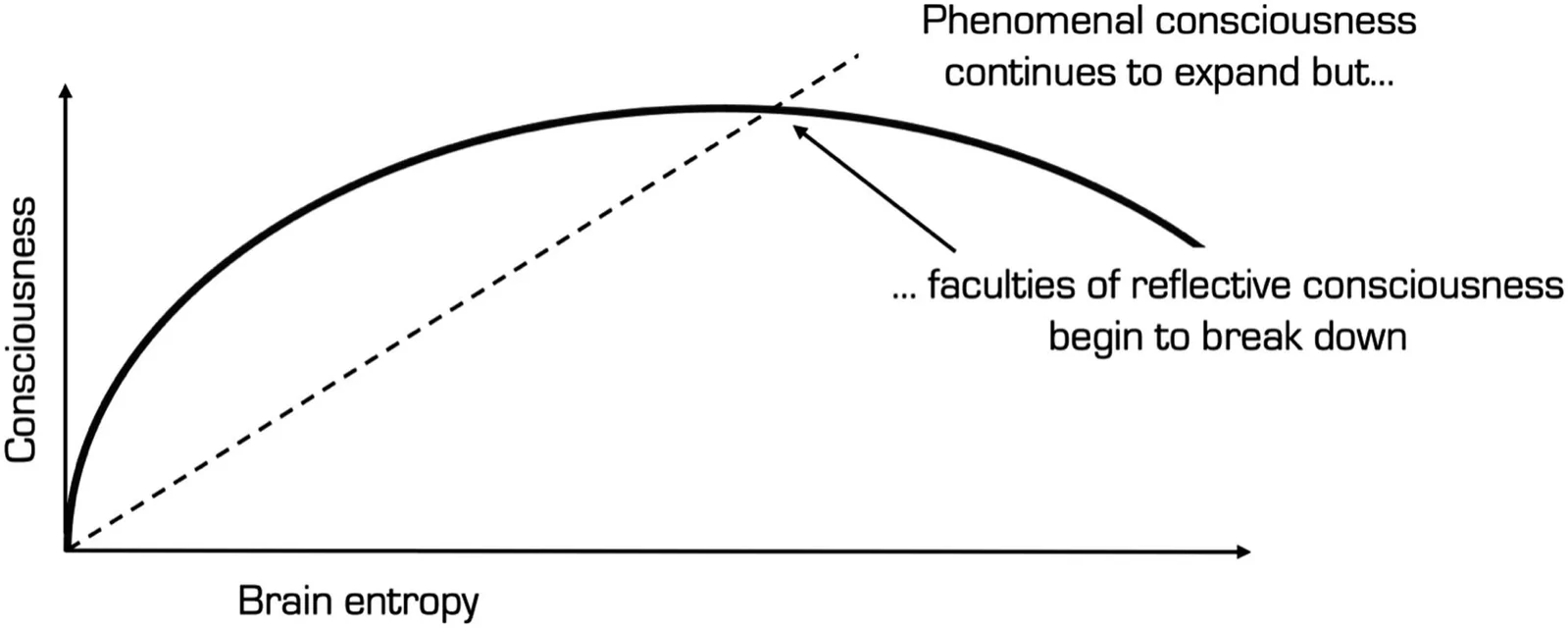
**The entropic brain is a model of the breadth or richness of phenomenal consciousness, also referred to as subjective experience**. Faculties of reflective consciousness may be compromised during states of extremely high brain entropy, while phenomenal consciousness is preserved. A profile of this (unusual) nature may occur with high-dose 5-MeO-DMT. 5-MeO-DMT = 5-methoxy-*N*,*N*-dimethyltryptamine.

### Summarizing the validity of the entropic brain hypothesis

The EBH has several strengths, including good convergent, correlative, predictive, discriminative and external validity, as summarized in Tables 2 and 3. Running through each, the EBH’s convergent validity is backed by the evidence from a broad variety of metrics of both temporal and spatial entropy—computed on data from different neuroimaging modalities. Its external validity is backed by changes in peripheral physiology and behaviour, pointing towards a consistent relationship with conscious experience, namely: entropy is elevated in expansive states and decreased in reduced states of consciousness. The EBH’s correlative or criterion validity is supported by a wealth of evidence, but some direct tests have also been cited, e.g. Timmermann *et al*.^12^ We also note that the EBH has very good face validity, in the sense that the bulk of its supportive evidence has been acquired in humans.

**Table 3:** Evidence for the discriminative, predictive and external validity of the EBH

Discriminative validity	Predictive validity	External validity/portability
Non-psychedelic but psychoactive control drugs fail to elevate brain entropy^38,62,79,83,86,197^	Entropy of spontaneous brain activity (ESBA) under psilocybin predicts improved well-being at 1 month^66,68^	EBH relevant to behaviour,^181,182^ proteomic signalling^77^ and peripheral physiology^178-180^
Operational definition/measure of IIT, perturbational complexity index was insensitive to psychedelic while LZc ‘was’ sensitive.^67^ Type 1 but not type 2 complexity increases with psychedelics^55,195,196^	ESBA under psilocybin predicts reduced precision weighting of negative self-beliefs^198^	EBH relevant to intelligence^155,183^ and artificial intelligence^199-202^
Brain entropy suppressed by goal-directed cognition but increased by sensory complexity^187-189^	ESBA under psilocybin predicts psychological insight^52,66,68^	EBH relevant to creativity and insight^52,66,68,149-154^
		EBH relevant to ageing^174-179^

EBH = entropic brain hypothesis; IIT = integrated information theory; LZc = Lempel-Ziv complexity.

One potential critique of the EBH relates to its previous emphasis on the ‘richness’ of subjective experience.^4^ For example, extreme meditative states, the near-death experience and the 5-MeO-DMT experience are ‘expansive’ but not necessarily ‘enriched’. Future work might aim to parse between expansive and enriched states, e.g. by closely comparing the phenomenology and brain effects of DMT with those of 5-MeO-DMT, where the former is characteristically ‘content rich’— whereas the latter is characteristically ‘expansive’. One possible reason for this difference may be that some examples of type 2 complexity (i.e. criticality) are preserved or exaggerated in the DMT state but compromised in the 5-MeO-DMT state.

The predictive validity of the EBH is nicely exemplified by the recent work of Lyons *et al*.,^66,68^ where a strong predictive relationship was found between the entropic brain effect (LZc on EEG) induced by high-dose psilocybin and subsequent improvements in well-being assessed 1 month after dosing (Fig. 2). Increased entropy in all three EEG recordings, at 60, 120 and 270 min post-dosing, predicted well-being improvements at 1 month. If such relationships prove reliable, this finding will represent the discovery of a valuable predictive biomarker of mental health improvements via psychedelic treatment. The work of Lyons *et al*.^66,68^ implies that the biomarker (increased brain entropy), which emerges in concert with the acute psychedelic experience, i.e. in a matter of minutes, has predictive validity for mental improvements several weeks later. In the same study, deeper analyses revealed that the relationship between the entropic effect and improvements in well-being are mediated by psychological insight, adding an explanatory, mechanistic richness to the model. Contrary to the (questionable) view that psychedelic therapy rests on mere expectancy effects,^203,204^ empirical work,^205^ including the above-cited mechanistic work of Lyons et al. implies that the main causal driver of therapeutic responses to psychedelic therapy is the induction of a state of psychological plasticity, mediated by the entropic brain effect, meshing with a supportive context. See Carhart-Harris *et al*.^93^ for a related discussion. According to this model, ‘plasticity’ and ‘contextual support’ combine synergistically to relax the weight of pathological habits of mind and behaviour that define the relevant illness.^185,198,206,207^

**Figure 2:**
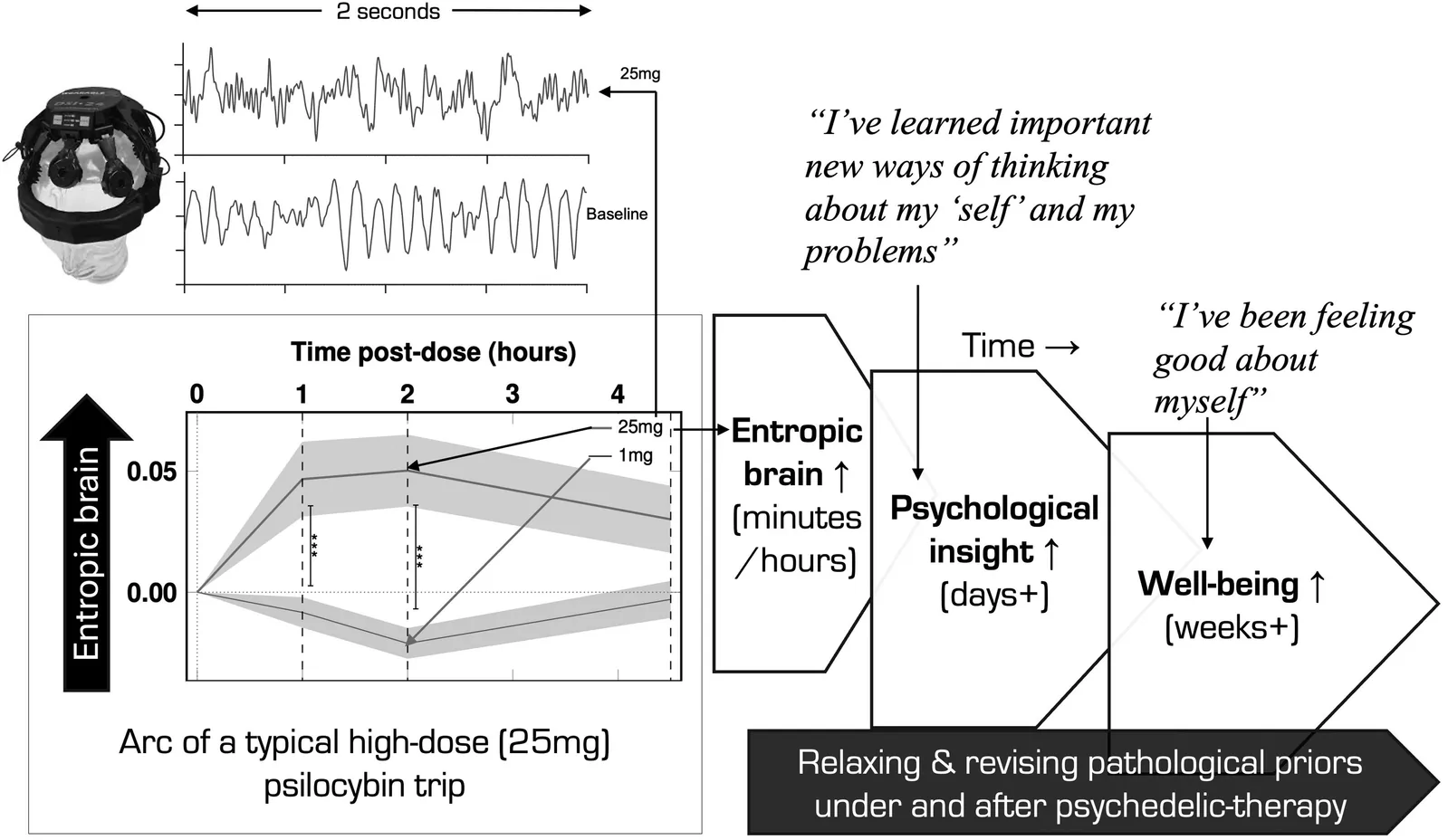
**The entropic brain in EEG predicting insight and improved well-being.**  *Top left*: Two seconds of EEG-recorded brain activity from an example subject. The *top* trace shows entropic activity versus the *bottom* trace, which was pre-dose baseline. *Bottom left*: The temporal profile of an entropic brain effect under 25 mg psilocybin, a high dose, averaged across 28 healthy volunteers. The arc of the effect matches the subjective effects of 25 mg psilocybin (oral), i.e. with peak effects occurring around 2 h post-administration. Within the same chart, we can see that an (inactive) 1 mg placebo dose of psilocybin failed to raised brain entropy, supporting assumptions regarding its inactivity and classification as a placebo. *Right*: The three-chevron schematic illustrates how an entropic brain effect—arising in minutes—predicts improvements in well-being several weeks later. This relationship is mediated by sub-acute psychological insight. Example items from an insight^228^ and well-being^229^ scale are shown. Notably, participants in this study—who were entirely naive to psychedelics when recruited—rated their high-dose (25 mg) psilocybin experience as the ‘single most’ unusual state of consciousness of their entire lives. See Lyons *et al*.^66,68^ for more details.

The discriminate validity of the EBH is supported by work showing that other psychoactive drugs—known to alter conscious experience—but not in an expansive fashion—do not increase brain entropy. Specific examples include methamphetamine,^62^ methylphenidate,^38^ the sedative deliriant scopolamine,^79^ other sedatives^83^ as well as the novel ‘nootropic’, piracetam.^197^

Furthermore, evidence favours the EBH versus an alternative theory of consciousness known as the ‘integrated information theory’ of consciousness or IIT.^208,209^ In a relevant study involving psilocybin, an operational measure of IIT—known as the ‘perturbational complexity index’ or PCI—was used. PCI involves applying a transcranial magnetic stimulation (TMS) pulse to the brain after which brain complexity is measured. Applying the PCI approach to individuals under the influence of psilocybin failed to yield an increase in this index of IIT/consciousness, whereas the classic LZc index (the go-to metric of the EBH)—applied to task-free resting-state conditions within the same study—did reveal the entropic brain effect of psilocybin.^67^ This finding addresses a previous critique of the EBH that applying a complexity measure (e.g. LZc) ‘depurated of [an] integration… term’ is a limitation of the EBH. On the contrary, simplicity is a notable strength of the EBH; it is a model that ‘explains much with little’. Indeed, one could even go so far as to say that the elusive ‘integration’ term of IIT currently appears not to be adding any extra explanatory power to what can be achieved with a simple measure of informational entropy applied to spontaneous brain dynamics, i.e. LZc. Some recent developments in information theoretic data analysis could yet change this,^27^ but the (putative) value of those metrics have yet to be fully established.

Briefly, in terms of the external validity or portability of the EBH, this is supported by work in generative artificial intelligence where an entropy or ‘temperature’ function is used to modulate the variability of outputs.^199,200^ We also note that entropic principles are being employed in AI and robotics, computer science more broadly and deep learning.^201,202^

The EBH has served as a bedrock for subsequent models to scaffold on. An obvious example is the ‘Relaxed Beliefs Under Psychedelics’ or ‘REBUS’ model, which leverages predictive processing,^210^ hypothesizing that increased brain entropy under psychedelics parallels a relative relaxation of the precision weighting of internal predictive models encoded by the regularities of high-level brain dynamics.^207^ Predictive processing maintains that an effective down-sampling (of experience) is operative during normal waking consciousness, whereas REBUS proposes that this breaks down under psychedelics, enabling more potential information to register in conscious experience. It is easy to see the parallels here with the EBH—which was the forerunner to REBUS.

Another model that has scaffolded on the EBH is ‘the canalization model of psychopathology’ or ‘canal model’ for short.^185^ The canal model leverages the construct of ‘canalization’—as it is formally defined in evolutionary science, i.e. as the process by which a phenotype becomes resistant to change. Canalization is the formal inverse of plasticity. In evolutionary science, ‘phenotypic plasticity’ refers to the sensitivity of a phenotype to environmental influence. The canal model adopts the position that there is a principal component of mental illness^211^ and suggests that (most) symptoms of mental illness develop via canalization—an (often implicit) selective entrenchment of maladaptive emotions, thoughts and/or behaviours. In addition to the atypically strong encoding of the relevant phenotype (e.g. a style of affect, cognition or behaviour), what determines whether it is considered ‘unhealthy’ or not, is whether it is dissonant with wider social norms and values. For example, a person who has developed an elite sports skill is typically considered healthy, whereas someone who has developed symptoms of treatment-resistant obsessive compulsive disorder, extreme phobic anxiety or a hard drug addiction (as just some examples) are considered unhealthy, even though some degree of canalization has taken place in each case. See Carhart-Harris *et al*.^93^ for a relevant discussion.

The canal model took inspiration from both REBUS and the EBH, as well as the observation that entropic brain activity under psychedelics tends to de-weight maladaptive self-schemas.^198^ This aligns with an implication of REBUS that psychedelics, especially when employed as part of psychedelic therapy, tend to de-weight the affective, cognitive or behavioural products of canalization that define a mental illness. That is, the canal model sees symptoms of mental illness as canalized phenotypes. It can be further speculated that the ‘de-weighting’ action of psychedelic therapy is in-keeping with the diffusion principle, i.e. an illustration of the second law of thermodynamics. According to this hypothesis, psychedelic therapy causes an ‘allostatic recalibration’, enabling a restoration of healthy inferential mechanisms, as discussed here.^171^ The critical dynamics conducive to such a recalibration are provided by the entropic brain effect. It is tempting to further speculate that this recalibration is felt by individuals and interpreted as intrinsically directional—as moving towards mental reintegration and health.^212^ One final speculation is that unhealthy mental and behavioural habits are selectively targeted by psychedelics because their origin or aetiology involved adversity-related (selective) sampling, including avoidance.^93^ Many mental illnesses appear to involve high-level cortical regions—where psychedelic’s main proteomic targets are, the serotonin 2A receptors^213^ This may explain why high-level beliefs are an especially sensitive target of psychedelic therapy.^214^

## Limitations of the entropic brain hypothesis

Constructive critiques of the EBH,^26^ REBUS^215,216^ and canal model^217^ have been proposed before, but it is beyond the remit of the present work to address each of them here. This said, it is necessary and appropriate to discuss some of the most important limitations of the EBH.

One critique is that several non-equivalent metrics of signal complexity have been ‘lumped together’ and treated as relevant to the EBH (e.g. potentially conflating type 1 and 2 complexity). It is accepted that certain type 2 complexity metrics may be insufficiently consistent with entropy. A more fine-toothed analysis is needed to ascertain which metrics, cited within the present work, legitimately reflect a signal with more ‘bits’ of potential information, i.e. a signal with high entropy, and which reflect changes in (e.g. type 2) complexity (or criticality) that are (at least somewhat) orthogonal to this.

Countering this critique, however, metrics of type 1 and 2 complexity often behave co-linearly, providing equivalently effective markers of the felt breadth of a given conscious state—because of a shared relevance to the EBH (i.e. the usefulness of type 2 complexity measures may be largely carried by entropy). This said, future extensions of the EBH may endeavour to better parse between type 1 and 2 complexity, showing how each relate to (different aspects of) consciousness, as discussed above. For clarity, it has felt necessary to commit to the position that it is specifically ‘information theoretic entropy’ (versus a signature of self-organized criticality or a type 2 complexity) that currently defines the EBH—including its (valuable) simplicity.

Lastly, comparable models to the EBH have been proposed before^218,219^ and since^220^ the original 2014 paper.^3^ Despite the existence of these complementary models, the EBH remains a sufficiently unique model of ‘conscious states’. First, the EBH is intentionally parsimonious. Unlike IIT or GNW, it does ‘not’ claim to be an explanatory theory of consciousness.^22,209,221^ These explanatory models aspire to explain how brain function gives rise to consciousness, i.e. they attempt to solve the ‘hard problem of consciousness’ in the (arguably problematic) terms in which it is posed.^222^ Instead, the EBH offers a ‘descriptive’ model of ‘conscious states’.^223^ It recognizes the legitimacy of causal complementarity between subjective experience and a relevant level of neurophysiology—meaning, the former can causally lead the latter (while remaining coupled to it). At the relevant scale, this bidirectional causality occurs without any decoupling between the essential components—mind, body and brain. These phenomena can be said to be ‘causally entangled’.

It is also appropriate to explicitly recognize that the EBH tends to avoid the term ‘consciousness’ itself. This is a deliberate omission that relates to the view that ‘consciousness’ is a vague, composite construct. The construct requires at least two of its main constituent components to be separately defined, one of which is ‘phenomenal consciousness’, and this is what the EBH commits to describing.

The EBH embraces expansive phenomenal states, whereas other models of consciousness generally do not (Table 1). Other models have tended to focus solely on the reduced consciousness end of the consciousness spectrum. This has led to a relatively impoverished (consciousness) science that has failed to leverage the special empirical and epistemic value of psychedelic drug research. The EBH is relatively unique in embracing the full spectrum of conscious states—including states that many people regard as being the most meaningful^224^ and unusual^66,68^ of their entire lives.

Lastly, if we are to tackle other major components of consciousness, such as ‘reflective awareness’ or ‘ego-consciousness’, then the need for an integration term may become increasingly pressing. Intriguing candidates may be found within the broad field of complexity science, and especially the sub-domains of ‘emergence’, ‘synergistic information processing’^27^ and ‘self-organized criticality’. One final speculation is that high-dose 5-MeO-DMT increases brain entropy beyond a zone of self-organized criticality into a regime of extremely high entropy, where ego-consciousness cannot hold but phenomenal consciousness goes on, albeit in a profoundly altered form, i.e. it is felt to be exceptionally expanded.

Lastly, the original entropic brain paper of 2014^3^ included a discussion of principles of depth psychology and psychoanalysis specifically. This perspective on the human mind remains of special interest. Its commitment to phenomenology—first-person experience^225^ is a special merit of depth psychology, which is courageous in looking beyond the limited scope of ego-consciousness—the quality of normal waking consciousness of healthy adult humans. Future work—in better-suited forums—may revive this topic and approach, to provide a richer, more poetic account of the mind than is appropriate here.

## Conclusions

All models are wrong but some are useful,^226^ for a while. Theoretical models are subject to the same evolutionary principles that govern the cosmos, not just the survival of certain species on our living planet.^227^ If a theory fails to adapt, it dies. Since its initial conception, the EBH has adapted and retained its value. Today, the model can serve to predict mental health breakthroughs,^66,68^ potentially in real-time,^72^ promising to inform on a paradigmatic challenge to the still dominant biocentric model of mental healthcare. It is hoped that others may feel inspired to leverage the EBH to guide their own philosophical, psychological, biological and medical research, while spotlighting where the model is weak and amenable to revision.
